# Exploration of Pattern Recognition Receptor Agonists as Candidate Adjuvants

**DOI:** 10.3389/fcimb.2021.745016

**Published:** 2021-10-06

**Authors:** Guang Han Ong, Benedict Shi Xiang Lian, Takumi Kawasaki, Taro Kawai

**Affiliations:** Laboratory of Molecular Immunobiology, Division of Biological Science, Graduate School of Science and Technology, Nara Institute of Science and Technology (NAIST), Ikoma, Japan

**Keywords:** adjuvant, PAMP, TLR, pattern recognition receptor (PRR), innate immunity

## Abstract

Adjuvants are used to maximize the potency of vaccines by enhancing immune reactions. Components of adjuvants include pathogen-associated molecular patterns (PAMPs) and damage-associate molecular patterns (DAMPs) that are agonists for innate immune receptors. Innate immune responses are usually activated when pathogen recognition receptors (PRRs) recognize PAMPs derived from invading pathogens or DAMPs released by host cells upon tissue damage. Activation of innate immunity by PRR agonists in adjuvants activates acquired immune responses, which is crucial to enhance immune reactions against the targeted pathogen. For example, agonists for Toll-like receptors have yielded promising results as adjuvants, which target PRR as adjuvant candidates. However, a comprehensive understanding of the type of immunological reaction against agonists for PRRs is essential to ensure the safety and reliability of vaccine adjuvants. This review provides an overview of the current progress in development of PRR agonists as vaccine adjuvants, the molecular mechanisms that underlie activation of immune responses, and the enhancement of vaccine efficacy by these potential adjuvant candidates.

## Introduction

A vaccine consists of immunogenic materials that help the host to acquire immunity against a pathogen. Vaccination confers protection to the host by initiating a dynamic immune response that mimics actual infection. Therefore, when the vaccinated host encounters the pathogen, the host immune memory mounts the specific immune response rapidly to eradicate the pathogen and prevent immune complications.

Historically, vaccine development has closely followed Pasteur’s principle of “isolation, inactivation, and administration” whereby the causative agent has to be identified, isolated, inactivated or attenuated, and finally administered to the host. With the advancement of innovative technologies, some vaccines have incorporated nanotechnology, which use novel nanoparticles to deliver components of the pathogen such as subunit proteins or nucleic acids. New technologies in vaccine development have also addressed the need for safe and negligible off-target effects. These novel agents offer many advantages such as high bioavailability, high throughput and time effectiveness in development, and safety relative to the conventional method. However, usually, these agents alone possess low reactogenicity and thus poorly induce immune responses.

Combining other materials, such as mineral particulates, plant derivatives, DNA oligodeoxynucleotides, and nanoparticle compounds, with a vaccine improves the vaccine’s overall efficacy to promote a robust immune response and maximize protection against the target pathogenic microorganism. These materials are known as adjuvants based on the Latin word *adjuvare* that describes the adjuvant’s role in “helping” potentiation of the vaccine. The US Food and Drug Administration (FDA) outlines an adjuvant as a constituent material of vaccines, which induces a specific immune response (Food and Administration, 1997). To date, very few adjuvants have been approved by the FDA that only supports an adjuvant’s use if it significantly improves the vaccine efficacy. Although several approved adjuvants are currently in use for vaccine development in humans, the mechanisms of adjuvants remain unknown.

## Innate Immunity

**Figure 1:**
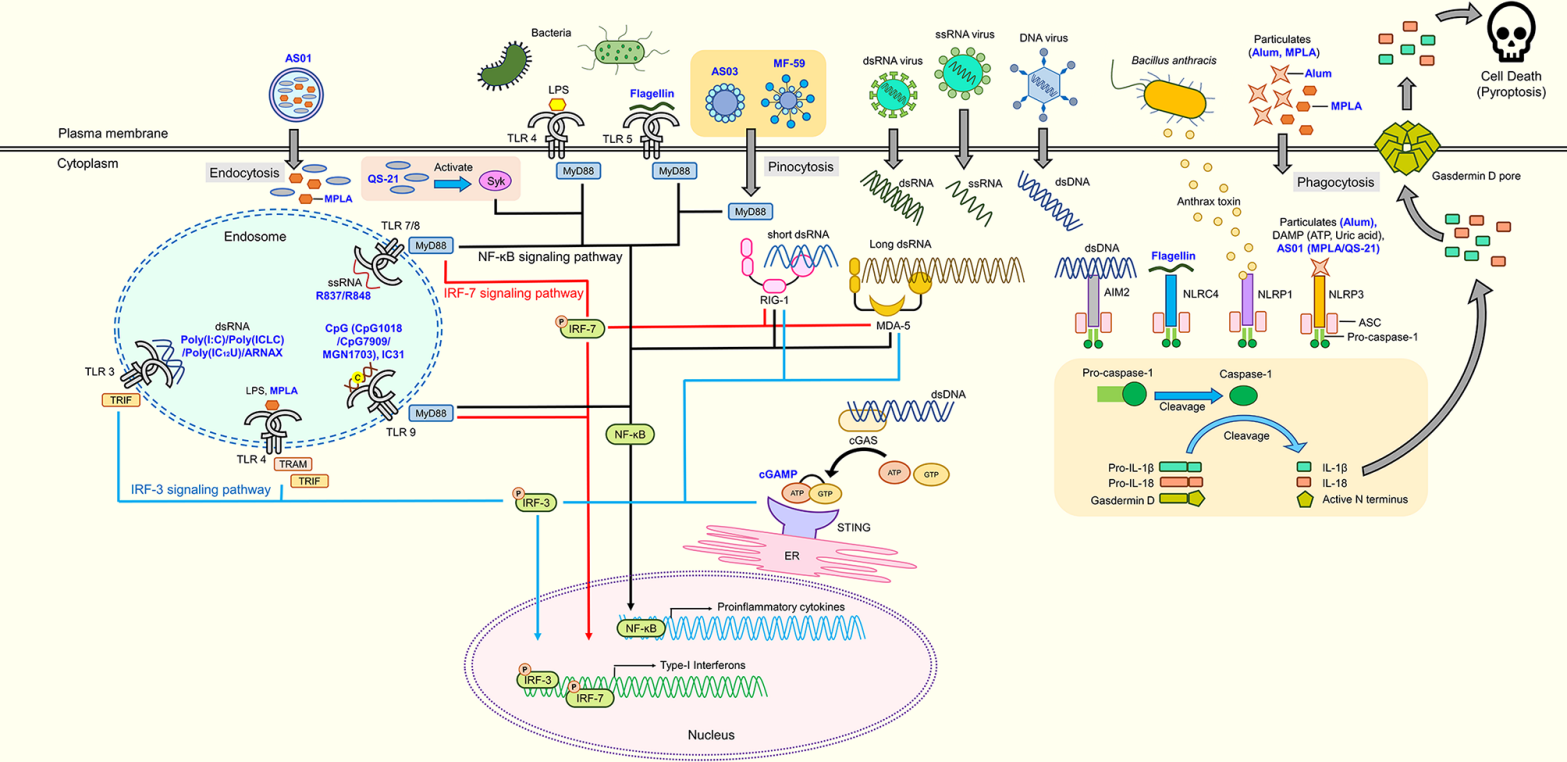
Adjuvant candidates and their respective receptors. TLR4 and TLR5 are expressed on the cell surface, while TLR3, TLR4, TLR7, TLR8, and TLR9 are expressed in endosomes. TLR4, TLR5, TLR7, TLR8, and TLR9 initiate signaling through the MyD88 pathway to activate NF-κB, which induces the production of proinflammatory cytokines. Activated Syk elicits the production of proinflammatory cytokines through the NF-κB signaling pathway. RIG-1 and MDA-5 are intracellular nucleic acid sensors that induce both proinflammatory cytokines and type-1 interferons *via* MyD88 and IRF signaling pathways. cGAS produces cGAMP that activates the cGAS-stimulator STING located on the ER to induce production of proinflammatory cytokines through the NF-κB signaling pathway. Inflammasomes (AIM2, NLRP1, NLRP3, and NLRC4) cleave pro-caspase 1 into caspase 1 that subsequently cleaves pro-IL-1β, pro-IL-18, and Gasdermin D into their mature forms, respectively. N-terminal Gasdermin D oligomerizes to form pores, inducing pyroptosis. Blue text represents adjuvant candidates discussed in this review.

Innate immunity plays an important role in the induction of host protection by vaccination. Innate immunity is the first line of defense in which the immune system recognizes the presence of pathogens or cellular damage (Akira et al., 2006). Innate immune cells possess sensors known as pattern recognition receptors (PRRs) that recognize pathogen-associated molecular patterns (PAMPs) released by pathogens or danger-associated molecular patterns (DAMPs) in the presence of damaged cells or tissues. Upon recognition, activation of innate immunity releases various cytokines or factors that initiate adaptive immunity. The first group of PRRs discovered and best characterized to date is the Toll-like receptor (TLR) family. Humans possess 10 TLRs, while 12 have been found in mice (Kawai and Akira, 2009). These TLRs are classified by their localization, namely plasma membrane or intracellular endosome TLRs. Each TLR is responsible for detecting specific PAMPs or DAMPs, as summarized in **Figure 1**. The location of TLRs is closely related to their functional properties and subsequent downstream signaling pathway. Plasma membrane-localized TLRs usually recognize pathogenic components such as proteins and lipid, whereas intracellular endosome TLRs detect nucleic acids (Barton and Medzhitov, 2003; Pandey et al., 2015). Lipoproteins are recognized by TLR1, TLR2, and TLR6, whereas lipopolysaccharide (LPS) and flagellin are recognized by TLR4 and TLR5, respectively. Nucleic acid materials, such as double-stranded RNA and single-stranded RNA, are detected by TLR3 and TLR7/8, respectively, and TLR9 recognizes single-stranded DNA. TLR4 localizes to the endosome after recognizing LPS. Therefore, similar to other endosomal TLRs, TLR4 induces two different downstream signaling pathways, depending on the location of TLR4 (Kawasaki and Kawai, 2014a). Upon detection of these signals by TLRs, they recruit Toll/Interleukin-1 receptor (TIR) domain-containing adaptor proteins, such as Myeloid differentiation primary response 88 (MyD88) and TIR-domain-containing adaptor-inducing interferon-β (TRIF), to initiate the downstream signal transduction pathways, activating Nuclear Factor kappa-light-chain-enhancer of activated B cells (NF-κB), interferon regulatory factors (IRFs) or mitogen-activated protein kinases (MAPKs) (Kawasaki and Kawai, 2014b). Subsequently, TLR activation leads to production of inflammation-related mediators and type I interferon (IFN).

In addition to TLRs, several cytosolic sensors collectively known as retinoic acid-inducible gene-I (RIG-I)-like receptors (RLRs) are responsible for detecting RNA species of invading pathogens in the cytoplasm (Loo and Gale, 2011). There are three members in the RLR family: RIG-I, melanoma differentiation-associated protein 5 (MDA5), and laboratory of genetics and physiology 2. RIG-I and MDA5 are the main receptors of cytosolic viral nucleic acids (Kato et al., 2006). Upon recognition, RLRs interact with the adaptor protein, mitochondrial antiviral-signaling protein (MAVS; also known as IFN-β promotor stimulator) to activate TANK-binding kinase 1 (TBK1) and IκB kinase-ε, phosphorylating interferon regulatory factor 3 (IRF3), and IRF7 to induce transcription of antiviral type 1 interferons (Hartmann, 2017b; Rehwinkel and Gack, 2020). Similarly, cyclic GMP-AMP (cGAMP) synthase (cGAS) is a cytosol DNA sensor that detects cytosolic DNA from bacteria, viruses, or even host DNA (Chen et al., 2016; Hopfner and Hornung, 2020). The binding of DNA to cGAS catalyzes synthesis of 2ʹ3ʹ-cGAMP that binds to the endoplasmic reticulum (ER) membrane adaptor stimulator of IFN genes (STING) (Ishikawa and Barber, 2008; Wu et al., 2013). Activated STING then migrates to the ER-Golgi compartment to recruit TBK1 that phosphorylates IRF3 to induce expression of type 1 IFNs (Tanaka and Chen, 2012; Dobbs et al., 2015).

Finally, certain sensors, such as nucleotide-binding oligomerization domain-like receptors (NLRs) or absent-in-melanoma-2 (AIM2)-like receptors, together with the adaptor protein apoptosis-associated spec-like protein containing a CARD (ASC) and caspase-1, form an intracellular multimeric protein complex known as the inflammasome to regulate the maturation and release of interleukin- (IL)-1β and IL-18 (Franchi et al., 2012). Examples of inflammasomes are nucleotide-binding domain, leucine-rich-containing family, pyrin domain-containing (NLRP1), NLRP3, NLR family CARD domain containing-4 (NLRC4), AIM2, and the pyrin family. These inflammasomes are activated by a wide range of PAMPs and DAMPs. NLRP1b in murine cells detects bacterial toxins (Levinsohn et al., 2012), NLRC4 is activated by components of type three secretion systems of gram-negative bacteria (e.g., flagellin) (Zhao et al., 2011), whereas AIM2 senses intracellular dsDNA (Hornung et al., 2009). Conversely, the NLRP3 inflammasome is activated by ionic influx, mitochondrial dysfunction, ROS production, and lysosomal damage that are induced by stimuli from pathogens or damaged host cells (Kelley et al., 2019). Upon stimulation, the sensor and ASC form a large protein complex that recruits pro-caspase-1, which causes cleavage and maturation of pro-caspase-1 into caspase-1. Caspase-1 then catalyzes cleavage of inactive pro-IL-1β and pro-IL-18 into their active forms (Rathinam and Fitzgerald, 2016). Additionally, caspase-1 is responsible for maturation of gasdermin D. Its mature form oligomerizes at the plasma membrane, which forms pores and subsequently causes pyroptosis (proinflammatory cell death) and the release of mature IL-1β and IL-18 (Liu et al., 2016).

Innate immunity provides a rapid reaction against invading pathogens. However, it lacks specificity and is unable to produce a long-term memory response. In the context of vaccination, adaptive immunity is crucial for the required specificity and provides long-term protective immunity against the recurring target antigen. Innate immunity provides the link to activate adaptive immunity. The secretion or induction of stimulatory molecules (CD40, CD80, and CD86), cytokines, and chemokines at the site of infection by activated cells in innate immunity aids in the recruitment and maturation of immune cells in adaptive immunity (Kang and Compans, 2009; Pulendran and Ahmed, 2011). Innate immune cells, such as dendritic cells (DCs) and macrophages, also act as antigen-presenting cells (APCs) to provide antigen-specific activation signals to adaptive immune T and B cells (Pasare and Medzhitov, 2004). Additionally, the types of cytokines released mediate different modes of T helper (Th) cell activation, namely Th1 or Th2 cell expansion. The Th1 response is mainly required to induce immunity against intracellular pathogens or tumors, whereas Th2 is primarily involved in eliminating extracellular pathogens by secreting antibodies (Korsholm et al., 2010). In brief, understanding how innate immunity works has greatly facilitated the development of effective adjuvants. In the following sections, we discuss licensed adjuvants, adjuvants in trials, and the innate immune responses stimulated by these adjuvant candidates.

## Currently Approved Adjuvants

Licensed adjuvants are grouped in accordance with their functional role in promoting vaccine efficacy. The first group of adjuvant acts as a vehicle that delivers the vaccine antigen. For example, alum salt (Glenny, 1926) and an emulsion (Freund, 1956) improve antigen presentation to APCs and recruit neutrophils and monocytes for rapid antigen transport to draining lymph nodes (Calabro et al., 2011). Conversely, the second group of adjuvants works as an immunostimulant by inducing the innate immune response. Liposome-based AS01 and CpG oligodeoxynucleotide exert immunostimulatory effects through TLRs. They activate innate immune responses in APCs. They also intermediate the adaptive immune response by acting as a potentiating agent of vaccine antigens to achieve synergistic engagement in host immunity.

### Alum

Aluminum salts, also known as alum, are the most common adjuvant in the vaccine industry. Alum is also the earliest adjuvant to be used in the 1920s when Glenny and colleagues found that purification of tetani and diphtheria toxoids with alum improves antibody responses in an animal model. Generally, alum compounds have different forms and may contain other elements such as sodium, cesium, and ammonium. However, alum in general refers to aluminum hydroxide and aluminum phosphate (Brito et al., 2013). Several activation mechanisms induced by alum have been proposed, although the actual mechanism has remained a subject of debate. Initially, it was believed that alum causes persistent and prolonged antigen release from the vaccine injection site, which is referred to as the “depot effect” (Glenny et al., 1926). This explanation was dismissed years later after some studies showed that removing the vaccine administration site did not affect the antibody titer of the specific antigen (Holt, 1950; Hutchison et al., 2012).

The surface of alum provides the capacity for the interaction with the antigen *via* several mechanisms such as electrostatic, hydrophobic, ligand exchanges, and many others. Protein antigens tend to be adsorbed to the solid surface at a pH near to the protein’s isoelectric point. The hydrophobic residues on the surface of protein antigens participate in the interaction with the surface of alum, which creates the adsorption effect. Furthermore, depending on the pH, the charge of alum promotes effective electrostatic interactions with various types of vaccine antigens. The positive surface charge of aluminum hydroxide and negative surface charge of aluminum phosphate at neutral pH promote electrostatic adsorption of antigens towards aluminum hydroxide or aluminum phosphate. Moreover, chemical modification by the ligand exchange mechanism, when the negative charge of the phosphate group terminal of antigen exchanges with the positively charged hydroxyl group of aluminum hydroxide, creates a strong interaction between alum and the antigen (Al-Shakhshir et al., 1994; Chang et al., 1997; Noe et al., 2010).

The interaction between alum and an antigen has been suggested to assist in delivering the antigen to APCs. Alums bind directly to lipids on the cell membrane of DCs because there is no specific receptor for alum (Flach et al., 2011). Additionally, alum forms particulates with soluble antigens *via* the adsorption effect, which facilitates uptake of the antigen by APCs *via* phagocytosis (Mannhalter et al., 1985; Ghimire et al., 2012). The phagocytosed alum-antigen causes lysosomal release of cathepsin B into the cytoplasm, which activates caspase-1-associated NLRP3 inflammasome activity (Hornung et al., 2008). Then, caspase-1 also catalyzes the production of proinflammatory cytokines such as IL-1β, IL-18, and IL-33 (Sutterwala et al., 2006; Eisenbarth et al., 2008; Li et al., 2008). Alum engaged by DCs also initiates several important signaling mechanisms that include phosphoinositide-3-kinase and calcineurin-nuclear factor of activated T cells (NFAT), which depends on spleen tyrosine kinase (Syk) (Mori et al., 2012; Khameneh et al., 2017). Moreover, alum-antigen promotes specialization of APCs that subsequently induce differentiation of naïve CD4(+) T cells to produce IL-4 and IL-5 for the Th2-type immune response (Rimaniol et al., 2004; Sokolovska et al., 2007). The cytotoxicity of alum also causes the production of several DAMPs, such as uric acid (Kool et al., 2008; Kool et al., 2011), ATP (Riteau et al., 2012), and heat-shock protein 70 (Wang et al., 2012), which serves as activation signals for the NLRP3 inflammasome.

Alum predominantly drives activation of the Th2 immune response, but a weak Th1 response. Th1 is not only responsible for protection against viruses and intracellular bacteria (Wang et al., 2012), but also facilitates cancer eradication (Granoff et al., 1997). Therefore, alum has poor adjuvanticity for vaccines against influenza (Granoff et al., 1997), malaria (Mata et al., 2013), and tuberculosis (Lindblad et al., 1997; Doherty and Andersen, 2005). The combination of alum with ligands of PPRs creates a balanced Th1 and Th2-type immune response. One of the earliest licensed adjuvants, AS04, combines alum with monophosphoryl lipid A (MPLA), an LPS derivative purified from *Salmonella minnesota* (Casella and Mitchell, 2008). Compared with LPS, MPLA has increased safety because it has lower reactogenicity than LPS. (Dowling and Levy, 2015). The phosphate groups on MPLA enable adsorption to alum by electrostatic interactions and ligand exchange, which allows the alum to acts as a vehicle to deliver MPLA to APCs and precisely activates the TLR4 response (Shi et al., 2001). Thus, compared with MPLA as a single agent, the addition of alum to MPLA induces rapid and robust production of proinflammatory cytokines *via* the TLR4 signaling pathway. The combination of alum with MPLA also prolongs cytokine induction and immune cell recruitment at injection sites (Didierlaurent et al., 2009).

The remarkable safety of alum makes it the first choice of adjuvant in vaccine research and development. To date, the use of alum alone or in combination has created many successful vaccines for humans, such as vaccines for tetanus (HogenEsch et al., 2018), diphtheria (HogenEsch et al., 2018), polio (HogenEsch et al., 2018), hepatitis A (HogenEsch et al., 2018), hepatitis B (AS04) (Kundi, 2007), human papillomavirus (AS04) (Harper et al., 2004; Harper, 2006; Paavonen et al., 2007), and the most recent SARS-CoV-2 (Wu et al., 2021; Xia et al., 2021; Zhang et al., 2021). Despite the significant advantages of alum, there are several drawbacks. The presence of alum often leads to swelling and erythema at the vaccine injection site, which is caused by strong innate and humoral immune responses such as excessive proinflammatory cytokines and antigen-specific IgE antibodies (Fawcett and Smith, 1984; Mark et al., 1995). Furthermore, *in vivo* models have shown aluminum element traces at the inoculation site even after 1 year post-administration (Gupta et al., 1996; Gupta et al., 1997), which pose a risk to induce severe chronic immune responses such as allergy (Netterlid et al., 2009). Moreover, alum stability at subfreezing temperature remains unresolved because alum salts form aggregates that significantly decrease the adjuvanticity of alum (Braun et al., 2009; Chen et al., 2009; Salnikova et al., 2012). Therefore, it is essential to prepare a formulation with alum limitations in mind to achieve maximum adjuvanticity and minimize side effects.

### Emulsions

The water-in-oil emulsion developed by Jules Freund in the 1930s was first used as an adjuvant. Freund found that a water-in-paraffin oil emulsion that contained dead Mycobacteria tuberculosis, later known as complete Freund’s adjuvant, initiates strong immunologic adjuvanticity and a high antibody titer (Freund et al., 1937; Freund, 1947). The use of Freund’s adjuvant has several drawbacks such as biodegradability of mineral oils, quality of the emulsifier, and formulation reproducibility (Murray et al., 1972; Shah et al., 2017). Moreover, the toxicity of the non-metabolized mineral oil and intolerable reactogenicities cause several local reactions that include granuloma formation and prolonged inflammation at the injection site (Lindblad, 2000). Systemic reactions such as anterior chamber uveitis have been reported in animal models (Waters et al., 1986; Allison and Byars, 1991). Later, incomplete Freund’s Adjuvant was developed by excluding killed mycobacteria and used in vaccine development for influenza (Salk et al., 1952; Bomford, 1980) and HIV (Silvera et al., 2004).

A different form of emulsion known as an oil-in-water emulsion was developed as an alternative. MF59, AS03, and AF03 are available and approved oil-in-water emulsions for use in human vaccines. These oil-in-water emulsions contain squalene and some other emulsion stabilizers, such as Span 85 (MF59), Tween 80 (MF59, AS03), and α-tocopherol (AS03) (Morel et al., 2011; Garçon et al., 2012; O’Hagan et al., 2013). These organic compounds have low viscosity, high biocompatibility, and can be fully metabolized in the human body without any safety issues (Vesikari et al., 2009). The MF59 oil-in-water emulsion was the earliest approved adjuvant applied in several influenza vaccines (H5N1 and H1N1) (Wilkins et al., 2017). MF59 was the first AS03 emulsion series reported to induce antibody production *via* the MyD88-dependent pathway without activating the TLR signaling pathway (Seubert et al., 2011). Loss of MyD88 in mice resulted in a significant reduction in antibody titer after immunization with an MF59-adjuvanted vaccine against *Neisseria meningitidis* (Seubert et al., 2011). Moreover, vaccinating mice that lack ASC, an adaptor protein within the NLRP3 inflammasome, with the MF59-adjuvanted vaccine against H5N1 influenza virus lowers the antibody titer (Ellebedy et al., 2011). However, antibody production in Nlrp3-knockout and Caspase 1-knockout mice remains intact after immunization with the MF59-adjuvanted H5N1 influenza vaccine (Ellebedy et al., 2011; Seubert et al., 2011). Therefore, antibody production after MF59-adjuvanted vaccination is proposed to be dependent on ASC, but independent of NLRP3 (Pulendran et al., 2021).

Similar to alum, MF59 and AS03 recruit APCs and granulocytes to the injection site (Seubert et al., 2008; O’Hagan et al., 2012). In fact, compared with alum, MF59 has a more prominent effect on recruiting neutrophiles and is more efficient in promoting antigen transport by myeloid cells to draining lymph nodes (Calabro et al., 2011). Furthermore, MF59 improves antigen uptake and presentation by monocytes to lymphocytes without a depot effect (Awate et al., 2013). Nevertheless, MF59 and AS03 are superior to alum for induction of cytokines, gene regulation, leukocyte migration, and antigen presentation (Dupuis et al., 2001; Mosca et al., 2008). MF59 upregulates expression of several cytokines (*Ccl2, Ccl4, Ccl5, Ccl12, Cxcl10, Il1b*, and *Il2*) much earlier than alum (Mosca et al., 2008). Moreover, MF59 induces higher cytokine expression than alum, such as *Tnf, Ccl17, Ccl24, Ltb*, and *Tgfb1* (Mosca et al., 2008). α-Tocopherol in AS03 regulates the production of some cytokines (e.g., CCL2, CCL3, and IL-6) and chemokines (e.g., granulocyte colony-stimulating factor and CXCL1) (Morel et al., 2011). Another study showed that MF59 also promotes excretion of extracellular ATP from muscle cells, which acts as a DAMP to initiate an innate immune response for cytokine production and NLRP3 inflammasome activation (Vono et al., 2013).

### Liposome-Based Immunostimulants

Liposome-based immunostimulants are a unique type of adjuvant encapsulated with a phospholipid bilayer known as a liposome (Copland et al., 2005). The only available licensed liposome-based immunostimulant is AS01. AS01 has been used to develop several vaccines that include vaccines against malaria (White et al., 2015), HIV (Leroux-Roels et al., 2010a; Van Braeckel et al., 2011) and tuberculosis (Leroux-Roels et al., 2013a). AS01 consists of two different immunostimulatory molecules, MPLA and QS-21, encapsulated in the liposome (Garcon and Van Mechelen, 2011). MPLA is the TLR4 ligand used in the AS04 adjuvant, whereas QS-21 is a triperpene glycoside saponin purified from fraction 21 of a bark extract from *Quillaja saponaria Molina* (Kensil et al., 1991). The liposome is used to deliver both MPLA and QS-21 into cells through cholesterol-dependent endocytosis (Welsby et al., 2017). QS-21 in the liposome induces lysosomal destabilization and later promotes tyrosine protein kinase SYK activation (Welsby et al., 2017). Moreover, MPLA in the cell induces the TRIF-dependent signaling pathway after binding to endosomal TLR4 (Watts et al., 2017). The single agent QS-21 is a potent compound with an undesirable hemolytic effect and induces cell death, which poses a tolerability issue in humans (Beck et al., 2015). The use of cholesterol-based liposomes abrogates the hemolytic activity of QS-21 and cell death induction (Marty-Roix et al., 2016).

MPLA and QS-21 of the AS01 adjuvant synergistically activate caspase-1 for NLRP3 inflammasome activation to release IL-1β as well as IL-18 from APCs, specifically subcapsular sinus macrophages (Detienne et al., 2016). The release of IL-18 by subcapsular sinus macrophages signals rapid and early production of IFNγ, especially by natural killer cells in the draining lymph node (Coccia et al., 2017). A high level of IFNγ results in maturation of DCs and induces a Th1-type immunity response. The synergistic effect of MPLA and QS-21 is canceled in the state of subcapsular sinus macrophage depletion, blocking IFNγ, and loss of NK cells, which suggests that AS01 initiates an IFNγ-dependent innate immune response (Coccia et al., 2017). Moreover, loss of TLR4 and caspase-1 significantly impacts the adjuvant effect of AS01. Lacking MPLA suppresses the expression of IL-1β because QS-21 alone is unable to induce IL-1β release, which suggests production of IL-1β induced by AS01 is dependent on TLR4 (Marty-Roix et al., 2016). Moreover, administration of either MPLA or QS-21 results in a minimal level of IFNγ^+^ NK cell induction (Coccia et al., 2017). Even though QS-21 activates caspase-1 for the NLRP3 inflammasome, deficiency in the Nlrp3 *in vivo* model does not affect the adjuvanticity of AS01 (Marty-Roix et al., 2016).

### Synthetic CpG Oligodeoxynucleotide (CpG-ODN) Immunostimulants

TLR9 specifically recognizes bacterial DNA motifs with the unmethylated cytosine-phosphate-guanine (CpG) dinucleotide for innate immunity activation through the MyD88-dependent pathway (Kawai and Akira, 2010). These immunostimulatory motifs have been adopted in synthetic adjuvant development with modifications to prevent nuclease degradation (Hanagata, 2012). CpG-ODN induces robust chemokine, cytokine, and antibody production in natural killer cells, B cells, and plasmacytoid DCs, causing a robust Th1-type immune response (Akira et al., 2006). Three classes of CpG-ODN ligands (classes A–C) have been developed, but only CpG-ODN from class B has been used in a clinical trial (Vollmer et al., 2004). These CpG ODNs have different nucleotide sequences and induce production of IFNα in plasmacytoid DCs (Verthelyi et al., 2001; Hemmi et al., 2003). CpG 1018, a licensed class B CpG-ODN (CpG-B ODN), is a monomeric oligonucleotide with high chemical stability and adjuvanticity to promote Th1-type immune responses (Campbell, 2017).

CpG-B ODN is engulfed and localizes to lysosome-associated membrane protein 1-expressing endosomes and causes maturation of plasmacytoid DCs through localization (Guiducci et al., 2006). Subsequently, when in the form of a microparticle, CpG-B ODN interacts with transferrin receptor 1-positive endosomes to enhance production of IFNα *via* the TLR9 signaling pathway (Guiducci et al., 2006). Moreover, CpG-B ODN interacts with B cells directly to stimulate antibody production (Hartmann, 2017a). A mouse model has shown that CpG-B ODN with antigen vaccination induces substantial and long-lasting antibody production compared with alum-adjuvanted or non-adjuvanted vaccines (Barry and Cooper, 2007). The recently licensed CpG 1018 is used as the adjuvant for hepatitis B vaccine Heplisav-B (JAMA, 2018). The use of the CpG 1018 adjuvant in Heplisav-B improves efficacy with only two dose regimens compared with conventional hepatitis B vaccines that require three dose regimens before achieving maximum protection (JAMA, 2018). At present, CpG 1018 is under clinical evaluation for several other vaccines that include vaccines against melanoma cancer (Speiser et al., 2005) and COVID-19 (Kuo et al., 2020).

## mRNA Vaccines and Adjuvants

Compared with conventional vaccines, nucleic acid vaccines have recently received much attention because of the application of the mRNA vaccine against severe acute respiratory syndrome-coronavirus 2 (SARS-CoV-2). mRNA vaccines have multiple advantages over conventional vaccines. For example, they are non-infectious, have no risk of insertional mutagenesis, are flexible for manipulation, and easy to produce (Pardi et al., 2018). Previously, it was challenging to develop truly usable mRNA vaccines because of the instability of mRNA and the lack of an efficient delivery method. Modern technology has resolved most of these problems using various delivery systems that also act as an adjuvant for mRNA vaccines.

Interestingly, mRNA is immunogenic by nature. The immunogenic property of mRNA is both beneficial and detrimental for mRNA vaccines. The presence of nucleic acid sensors, such as TLR3, TLR7/8, RIG-I, and MDA-5, recognize vaccinated mRNA, which then proceed to activate innate immune signaling pathways to produce type-I IFNs (Okude et al., 2020). As mentioned previously, activation of innate immunity is crucial for the development of adaptive immunity against the intended vaccine target. However, excessive production of type-I IFNs can lead to elevated activation of Eukaryotic initiation factor 2-α kinase that inhibits protein translation (de Haro et al., 1996), degradation of RNA, and induction of apoptosis (Liang et al., 2006; Garcia et al., 2007). Consequently, this will result in a reduced expression of the target antigen and thus lower the efficacy of mRNA vaccines. Furthermore, Pollard *et al*. demonstrated that expression of antigen-encoding mRNA is higher in TRIF knockouts, which is in agreement with previous studies showing that type-I IFNs may reduce mRNA vaccine efficacy (Pollard et al., 2013).

To deceive nucleic acid sensors, several strategies have been used. One method employed by BioNTech/Pfizer and Moderna in their approved SARS-CoV-2 vaccines is replacement of pseudouridine-incorporated mRNA with naturally occurring 1-methylpseudouridine to avoid detection by PRRs (Buschmann et al., 2021). This modification enhances the stability, expression, and translation of the target mRNA (Kariko et al., 2008; Andries et al., 2015). However, notably, adjuvants are not mentioned in the formulations of both vaccines by BioNTech/Pfizer or Moderna (FDA, 2020a; FDA, 2020b). BioNTech/Pfizer mentioned RNA to have adjuvant effects in their clinical study (Mulligan et al., 2020). The modified mRNA is delivered using liposome-based nanoparticles (LNPs). It is possible that the LNPs used by these vaccines act as both the delivery agent and adjuvant. Studies by Moderna suggested that activation of robust innate immunity may not be a prerequisite for successful immunization using an mRNA vaccine, which suggested that mild or modest activation of innate immunity by the LNPs in these mRNA vaccines is sufficient to provide the required adjuvanticity (Liang et al., 2017; Hassett et al., 2019).

An mRNA vaccine can be coupled with different formulations to increase vaccine immunogenicity. These formulations often target various PRRs of innate immune components. An example is TriMix in which the antigen mRNA is codelivered with a mixture of three other mRNAs that encode CD70, CD40 ligand, and constitutively active Toll-like receptor 4 that targets the TLR4 pathway. This formulation is currently in vaccine trials for HIV and cancer (Wilgenhof et al., 2016; Guardo et al., 2017). Another innovative strategy involves codelivering the antigen mRNA with an immunogenic RNA complexed with protamine (RNActive by Curevac) or other cationic peptides (RNAdjuvant by CureVac) that are often used to stabilize RNA. The stabilized RNA functions to induce activation of innate immunity in humans and mice *via* stimulation of RIG-I, MDA-5, and TLR7/8 receptors (Fotin-Mleczek et al., 2011; Kallen et al., 2013; Edwards et al., 2017; Ziegler et al., 2017).

## TLR Agonists as Adjuvant Candidates

A classic example of fundamental discoveries translated into an application is when researchers harnessed the ability of TLR agonists to initiate the innate immune system to be used as adjuvant candidates to improve the immunogenicity of vaccines. As mentioned in the previous sections, the discovery of TLR4-LPS led to the approval of a detoxified derivative of LPS, namely MPLA to be used as an adjuvant or a component of combined adjuvants. The subsequently approved TLR agonist adjuvant is CpG 1018, a TLR9 agonist. Since their initial licensing, these TLR agonists have also been studied as potential adjuvants in other vaccines such as cancer and allergen vaccines. In the following section, we will introduce several other adjuvant candidates in development, which are categorized in accordance with their corresponding TLRs.

### TLR3 Agonist

TLR3 is localized in the endosome of a cell, which primarily detects viral dsRNA. This receptor plays an important role in inducing an antiviral response and may be crucial to induce adaptive immune responses by stimulating conventional DCs (cDCs) for cross-priming (Datta et al., 2003; Schulz et al., 2005). Even before the discovery of TLRs, it was found that a synthetic dsRNA, polyriboisosinic:polyribocytidylic acid [poly(I:C)] is highly capable of inducing IFN production (Field et al., 1967). Together with RIG-I and MDA5, TLR3 recognizes poly(I:C) that mimics viral RNA to induce type I IFN, type III IFN, and Th1 cytokine responses (Okahira et al., 2005; Kato et al., 2006; Longhi et al., 2009). Upon recognition of poly(I:C) by TLR3, production of type I IFN by cDCs is particularly critical for cDCs to effectively cross-present antigen for subsequent CD8 T cell responses (Schulz et al., 2005; Durand et al., 2006). Additionally, the type I IFN response induced by poly(I:C) promotes clonal expansion of T cells, increases the effector T cell ratio, and effectively increases the numbers of antigen-specific B cells (Kolumam et al., 2005; Ngoi et al., 2008; Perret et al., 2013). Hence, there has been interest in investigating poly(I:C) as a potential adjuvant. However, the use of poly(I:C) alone may be too toxic in humans (Cornell et al., 1976; Robinson et al., 1976). Therefore, derivatives of poly(I:C), such as poly(ICLC) and poly(IC_12_U), and alternative synthetic TLR3 agonists (e.g., ARNAX, IPH 3102, and RGC100) have been developed and are currently under investigation to be used as adjuvants for infectious diseases and cancer.

Poly(ICLC) contains poly-L-lysine in carboxymethylcellulose and retains interferon-stimulating properties, but is more resistant to serum nucleases, thereby enhancing its immunostimulatory effect (Levy et al., 1975). Moreover, the addition of poly-L-lysine and carboxymethylcellulose has been suggested to assist transport of poly(I:C) into the cytosol and escape from the endosome, which potentially activates both TLR3 and MDA5 pathways (Sultan et al., 2018). An intriguing feature of poly(ICLC) is that, in addition to type I IFN, it induces expression of various other genes in the innate immunity pathway, including NF-κB pathway-related genes, components of the inflammasome, and the complement system, which is similar to the responses induced by live viral vaccines (Caskey et al., 2011). To date, poly(ICLC) has been used in adjuvant candidate studies of vaccines against infectious diseases, such as *Plasmodium falciparum* (Kastenmuller et al., 2013) and HIV (Flynn et al., 2011), and cancer (Ohlfest et al., 2013). Compared with other TLR agonists, such as LPS and CpG, poly(ICLC) has been demonstrated to induce a higher Th1 immune response that is more favorable in vaccination (Sabbatini et al., 2012).

Poly(IC_12_U) was explicitly designed to reduce the toxicity of poly(I:C) by having mismatched uracil and guanosine residues (Gowen et al., 2007; Engel et al., 2011; Martins et al., 2015). However, the reduced toxicity resulted in a lower type I IFN reaction than poly(I:C) (Gowen et al., 2007). Nonetheless, poly(IC_12_U) still enhances the immunogenicity of an H1N1 influenza vaccine in mouse and primate models (Ichinohe et al., 2009; Ichinohe et al., 2010). In contrast to poly(I:C) and poly(ICLC), poly(IC_12_U) appears to bind exclusively to TLR3, but not MDA5 (Gowen et al., 2007). A study conducted by Inochihe *et al.* showed that the use of poly(IC_12_U) with an antigen increases the transcription expression of RIG-I (Ichinohe et al., 2009). Similar to poly(ICLC), poly(IC_12_U) has been assessed in adjuvant studies of vaccines against HIV (Flamar et al., 2015), influenza (Overton et al., 2014), and cancer (Navabi et al., 2009).

Another emerging TLR3 agonist with an adjuvant potential is ARNAX, a TLR3-specific ligand synthesized intentionally with reduced toxicity relative to poly(I:C) (Seya et al., 2016). The toxicity of poly(I:C) originates from activation of the MAVS pathway (activation of RIG-I and/or MDA5) (Matsumoto et al., 2015). Hence, Matsumoto *et al*. developed a ligand that included GpC phosphorothioate oligodeoxynucleotides and dsRNA, which is internalized into the endosome for recognition by TLR3. Owing to the relatively short length of the RNA chain (140), the ligand activates TLR3 while avoiding detection by MDA5 (Matsumoto et al., 2015). In a mouse model, the ligand did not cause a significant increase in serum inflammatory cytokines, but facilitated cross-presentation of the antigen by DCs and induced a Th1-skewed profile (Takeda et al., 2017). ARNAX is mainly being studied for cancer immunotherapy (Matsumoto et al., 2020) and influenza vaccination (Takeda et al., 2018).

### TLR7/8 Agonists

TLR7 and TLR8 have emerged as attractive adjuvant candidates because studies have found that agonists of TLR7/8 highly induce the required Th1 immune response, which favors vaccination (Ghosh et al., 2006; Kwissa et al., 2012). Activation of TLR7/8 induces high levels of type I IFN, IL-12, TNF-α, and IL-1β. Among TLR agonists, TLR8 agonist is a strong inducer of the Th1 immune response (Ghosh et al., 2006). Additionally, TLR7/8 and TLR9 agonists are the only agonists that activate and induce expansion of both cDCs and plasmacytoid DCs, while also mobilizing CD14^+^CD16^+^ (inflammatory monocytes) and CD14^dim^CD16^++^ (patrolling) monocytes (Kwissa et al., 2012). In particular, a class of synthetic small molecules, imidazoquinolines, is frequently investigated for their potential as adjuvant candidates with imiquimod (R837) and resiquimod (R848) as the main representatives and several other TLR7/8 agonists patented and currently under study (Kieffer et al., 2020).

R837 is currently approved to treat genital warts, superficial basal cell carcinoma, and actinic keratosis, and R848 is actively being tested for antiviral and antitumor therapeutic use. However, trials of imidazoquinolines as adjuvant candidates have had mixed results because these small molecules have some intrinsic drawbacks. Specifically, they often diffuse from the site of application and thus away from the antigen, thereby lowering efficacy, and induce systemic side effects (Vasilakos and Tomai, 2013). Therefore, a specific method to deliver or direct conjugation of small molecules to the antigens is required to improve vaccine efficacy. Direct conjugation of TLR7/8 agonists to HIV-1 Gag protein or even whole inactivated influenza virus particles has increased Th1 responses and the number of antigen-specific T cells (Wille-Reece et al., 2005; Oh and Kedl, 2010; Kastenmuller et al., 2011; Holbrook et al., 2018). Some other conjugate formulations include intentional design of conjugate formulations that forms large particulates. Conjugation to synthetic polymer scaffolds, nanogels, lipid–polymer amphiphiles, alum, polyethylene glycol (PEG), or various other synthetic polymers has significantly improved delivery of TLR7/8 agonists, and increased mature DCs and antigen-specific T cells [extensively reviewed in (Bhagchandani et al., 2021)]. Another interesting attempt to deliver TLR7 agonists is using an oxidation-sensitive polymersome based on PEG-b-PPS reported by Scott et al. (2012). This method enabled the delivery of the tested TLR7 agonists (gardiquimod and R848) to the endosome to interact with endosomal TLR7, followed by a second later phase whereby the antigen escaped from the endosome, which transported the antigen to the cytosol, subsequently allowing the processing and presentation of the antigen by MHC I in DCs. Moreover, several other studies that employed a combination of TLR7/8 agonists with one or more TLR agonists, such as MPLA (TLR4) and MPLA + CpG ODN (TLR4 and TLR9), in which the combinations enhanced innate immunity responses, showed a significant surge of antigen-specific neutralizing antibodies and improved Th1 responses (Rizwan et al., 2013; Fox et al., 2014; Moody et al., 2014; Goff et al., 2015). All of these innovations demonstrate the potential of TLR7/8 agonists as adjuvant candidates.

### TLR9 Agonists

As mentioned in the previous section, a TLR9 agonist CpG 1018 is commercially available as an adjuvant in the Heplisav-B vaccine. Another CpG ODN, CpG 7909, has also entered clinical trials and showed promising results in HBV and malaria vaccination (Cooper et al., 2005; Ellis et al., 2012). Concurrently, other researchers have developed next-generation TLR9 agonists. A promising agonist is MGN1703, a small DNA molecule that also includes CG motifs, but is structurally distinct from CPG ODN. MGN1703 contains a stretch of reverse complementary DNA that is double-stranded in the midsection and flanked by two single-stranded loops that comprises three effective non-methylated CG motifs, thereby forming a dumbbell-shaped structure in contrast toCpG ODN that is linear (Kapp et al., 2014). Initially tested as an immunotherapeutic agent against cancer, MGN1703 proceeded to adjuvant trials and found to activate both innate and adaptive immune responses with only mild or transient side effects (Weihrauch et al., 2005; Kapp et al., 2014; Wittig et al., 2015).

Another interesting TLR9 agonist in human trials is IC31, a two component adjuvant with a synthetic antimicrobial peptide KLK and CpG-free TLR9 agonist ODN1a (Kritsch et al., 2005). IC31 produces a balanced response between NF-κB and IRF3 signaling pathways and stimulates DCs to enhance T cell proliferation, which leads to a Th1 response (Schellack et al., 2006; Bernardo et al., 2011). Several vaccine trials of IC31 as an adjuvant in humans for tuberculosis are currently ongoing (Mearns et al., 2017; Norrby et al., 2017; Hussein et al., 2018; Suliman et al., 2019) with potential use being considered in a dengue fever vaccine (Bernardo et al., 2011).

### TLR5 Agonist

TLR5 is expressed by various immune cells to engage and recognize bacterial flagellin, and then elicits downstream inflammation pathways to trigger the release of multiple inflammatory mediators such as TNF-α, IL-1, IL-6, and nitric oxide (Hayashi et al., 2001). However, as a ligand, flagellin evokes mixed Th1 and Th2 responses instead of Th1-biased responses induced by other TLR ligands (Huleatt et al., 2007). Additionally, flagellin activates the NLRC4 inflammasome to process and release IL-1β (Franchi et al., 2006; Zhao et al., 2011). Several studies have demonstrated that flagellin induces an adjuvant response in a TLR5- or NLRC4-independent model, albeit with lower efficiency than the wildtype. The adjuvanticity is greatly diminished when both TLR5 and NLRC4 are absent in the mouse model, which indicates that at least one of the sensors is required to elicit an immune response or both to reach optimal stimulation (Vijay-Kumar et al., 2010; Lopez-Yglesias et al., 2014). Furthermore, flagellin has been suggested to be an adjuvant in immunocompromised patients because flagellin could activate inflammasome *via* the NLRC4 pathway in DCs with NLRP3-defect isolated from HIV patients (Dos Reis et al., 2019).

Flagellin can be used as an adjuvant by several methods that are collectively reviewed by Cui et al. (2018). The simplest method is direct administration with an antigen. Notably, this delivery method successfully induces a mucosal immune response that is central in defense against respiratory and gastrointestinal infections (Lee et al., 2006; Hong et al., 2012). Moreover, flagellin is flexible for modification. Attempts to construct chimeric flagellins or coexpression of flagellin-antigen in live attenuated bacterial strain and generation of recombinant flagellin-antigen fusion proteins to be used as adjuvant vaccines for infectious diseases and tumors have all achieved certain degrees of success in animal models (Cui et al., 2018). To date, at least three vaccines that employ flagellin are in the clinical trial phase. Two vaccines target the influenza virus and one targets *Yersinia pestis*, a gram-negative bacterium that causes plague (Talbot et al., 2010; Treanor et al., 2010; Hong et al., 2012).

## STING Agonists

STING plays a central role in the activation of several cytosolic nucleic acid sensing pathways. For example, upon detection of cytosolic nucleic acids derived from pathogen, tumors or host by cGAS, cGAMP is synthesized, binds and activates STING to elicit the downstream pathways. Various studies have listed STING agonists as a therapeutic agent in cancer immunotherapy. Nevertheless, the development of STING agonists as adjuvants remains slow and, to the best of our knowledge, none have yet entered the human trial phase. Several studies in mice have shown that administration of these agonists improves antigen-specific Ab production, activates STING- and type I IFN-dependent responses, induces a balanced Th1/Th2/Th17 response, increases the duration of T cell responses, and prolongs mouse survival (Yan et al., 2009; Ebensen et al., 2011; Barker et al., 2013; Chandra et al., 2014; Skrnjug et al., 2014; Ahn et al., 2018; Kinkead et al., 2018; Sallets et al., 2018). TBK1- and IRF3-dependent, and possibly AIM2-mediated responses elicited by these agonists are important for adjuvant effects (Ishii et al., 2008; Ishikawa et al., 2009; Suschak et al., 2015). Notably, cGAMP—a natural STING agonist—has been reported to be superior as an adjuvant for intradermal influenza vaccination compared with intramuscular administration in mice and swine skin models, and thus can potentially be used as an adjuvant in cutaneous vaccination (Wang et al., 2016).

## Discussion

Throughout the years, vaccines have developed from the use of the whole antigen (killed or attenuated pathogens) to a minimalist approach in designing vaccines (subunit proteins or nucleic acid vaccines) from prevention of infectious diseases to anti-tumor therapy. However, the conceptualized minimalist vaccines usually lack the ability to stimulate host immune responses to produce the immunogenicity required against the target antigen, which raises the importance of adjuvants in vaccine formulations. Adjuvant development for humans is described as the slowest process in medicine history, despite the success of vaccine development in medical history. (De Gregorio et al., 2008). Adjuvant availability remains limited mainly because of the limitation in providing adequate support in boosting vaccine efficacy for a specific illness. Furthermore, vaccine strategies that only target adaptive immunity to initiate immunologic memory neglect other important immunological factors needed to boost vaccine efficacy. Fortunately, the emerging concept of innate immunity, which shapes the adaptive immune response, has refurbished the mechanism-of-action of vaccine adjuvants (Fearon and Locksley, 1996). In the previous sections, we have extensively discussed various types of adjuvant candidates and the activated innate immune responses (**Table 1**).

**Table 1:** PRR adjuvant candidates and their activation mechanism.

Adjuvant/Candidate	PRRs	Mechanisms	References
**Licensed adjuvants**			
Alum	NLRP3	Enters the cells *via* phagocytosis Activates NLRP3 inflammasome to induce the cleavage of pro-caspase 1 into caspase 1 Induces production of DAMPs to activate NLRP3 inflammasome	[40,41,42]
MF59, AS03	–	Enter the cells *via* pinocytosis Activate NF-κB dependent pathway *via* MyD88 without activating TLR signaling pathway	[93]
MPLA (AS01)	TLR4	Enter the cells *via* endocytosis Induces mainly IRF-3 signaling pathway *via* TRIF Activate NLRP3 together with QS-21	[60, 110, 112, 113]
QS-21 (AS01)	–	Enter the cells *via* endocytosis Activate Spleen tyrosine kinase (Syk) Induces NF-κB signaling pathway Activate NLRP3 together with MPLA	[109, 112, 113]
CpG 1018	TLR9	Induces NF-κB signaling pathway *via* MyD88	[121]
**Potential adjuvants**			
dsRNA (Poly (I:C), Poly (ICLC), Poly (IC12U), ARNAX)	TLR3	Induces mainly IRF-3 signaling pathway *via* TRIF	[9, 158, 165, 173]
ssRNA (R837/R848)	TLR7, TLR8	Induces NF-κB signaling pathway *via* MyD88	[178, 179]
CpG ODN (CpG7909, MGN1703), IC31	TLR9	Induces NF-κB signaling pathway *via* MyD88	[192, 193, 194, 197]
Flagellin	TLR5, NLRC4	Induces NF-κB signaling pathway *via* MyD88 Also activates NLRC4 inflammasome to induce the cleavage of pro-caspase 1 into caspase 1	[216, 218]

Vaccine development for HIV in the early 1990s revealed that the use of a single adjuvant may not be sufficient to elicit the required immune responses against pathogens. Therefore, several strategies are implemented to achieve a synergistic outcome of adjuvants in the vaccination model, especially to elicit proinflammatory cytokines for the innate immune response (Nakayama, 2016). Previous attempts to use a combination of multiple PRR adjuvants with recombinant hepatitis B surface antigen formulated together with an emulsion or liposomes showed encouraging outcomes (Vandepapelière et al., 2008). More trials have been conducted to explore possible combinations with high adjuvanticity through adjuvant synergism. For example, the AS15 adjuvant is a liposome-based adjuvant that includes MPLA, CpG ODN, and QS-21. A vaccine containing the AS15 adjuvant induces high antibody production and robust T cell activation compared with other adjuvants such as AS02B that lack CpG ODN (Kruit et al., 2013). Moreover, AS15 has other unique features such as a antineoplastic effect (Garçon et al., 2016). Therefore, AS15 is used in anticancer vaccine development (Kruit et al., 2013). A combination of multiple classes of PRRs in vaccines against HIV and TB also displays low reactogenicity, but retains high immunogenicity and less intolerable adverse effects such as fever and inflammation at the administration site, which indicate that the safety and tolerability of adjuvants in combination systems are approaching the ideal state of adjuvants (Leroux-Roels et al., 2010b; Leroux-Roels et al., 2013b).

Despite the popularization of minimalist vaccines, another rising concept in immunity cannot be ignored, namely trained innate immunity (Netea et al., 2011). Vaccination against bacilli Calmette-Guerin results in protection against heterologous infections (Goodridge et al., 2016), which implies that innate immunity can be trained or primed to provide cross protection against infections other than the intended pathogen. Several TLR agonists have been demonstrated to confer cross protection [reviewed by Sanchez-Ramon et al. (2018)]. The incorporation of these agonists into adjuvant formulations may confer protection against multiple infectious agents in a single vaccine.

Safety is another challenge of adjuvant development. Previously, alum and emulsions were postulated to cause chronic toxicity and inflammation because these adjuvants form long-term tissue depots (Petrovsky, 2015). Regrettably, alum and emulsions cannot be eliminated from an adjuvant formulation because their absence significantly reduces vaccine efficiency (Calabro et al., 2011). Formulating alum or emulsions with PRR agonists may reduce the amount of alum or emulsion needed, thereby minimalizing toxicity while maintaining a robust innate immune response. However, precautionary measures are crucial for those with immunodeficiencies, such as children and older individuals (Pasquale et al., 2015). Nonetheless, a broad selection of PRRs and a better understanding of their signaling pathways allow the development of targeted adjuvants by avoiding specific signaling pathways that may be detrimental to immunodeficient people (Vasou et al., 2017).

## Conclusion

From the licensed and potential adjuvants discussed in this review, it is evident that innate immunity is the critical determining factor for the efficacy of an adjuvant. Adjuvants that induce effective innate immunity have a high possibility to boost vaccine efficiency and achieve full protection against diseases. Thus, the ability to initiate an innate immune response should be highlighted as the principal criterion for a potential adjuvant. PRR agonists have received attention as future adjuvant candidates because of their ability to promote a potent innate immune response. In the foreseeable future, we should expect the use of more PRR agonists in adjuvant formulations for clinical trial. Despite tremendous progress in searching for novel adjuvants for vaccine development in recent decades, further research must broaden the adjuvant varieties, which are suitable for all individuals with safety and vaccine efficacy.

## Author Contributions

GO and BL wrote the manuscript. TK edited and TKawai supervised the manuscript. All authors contributed to the article and approved the submitted version.

## Conflict of Interest

The authors declare that the research was conducted in the absence of any commercial or financial relationships that could be construed as a potential conflict of interest.

## Publisher’s Note

All claims expressed in this article are solely those of the authors and do not necessarily represent those of their affiliated organizations, or those of the publisher, the editors and the reviewers. Any product that may be evaluated in this article, or claim that may be made by its manufacturer, is not guaranteed or endorsed by the publisher.
